# Study protocol for a 2-year longitudinal study of positive youth development at an urban sport for development facility

**DOI:** 10.1186/s12889-019-7843-5

**Published:** 2019-11-08

**Authors:** Marika Warner, Gillian White, Jackie Robinson, John Cairney, Jessica Fraser-Thomas

**Affiliations:** 1 MLSE LaunchPad, 259 Jarvis Street, Toronto, ON M5B 2C2 Canada; 2 University of Toronto, Faculty of Kinesiology & Physical Education, 55 Harbord Street, Toronto, ON M5S 2W6 Canada; 3 York University, Faculty of Health, 170 Campus Walk, North York, ON M3J 1P3 Canada

**Keywords:** Sport for development - positive youth development - youth sport, Children, Adolescents

## Abstract

**Background:**

Youth facing barriers created by social marginalization are at a greater risk of adverse health outcomes, lower educational and occupational attainment, and decreased overall quality of life as adults. The negative psychosocial and physiological consequences of significant barriers to positive development during youth may be mitigated by interventions promoting physical activity, psychosocial development, and healthy behaviours. Sport for Development programming is a possible opportunity for youth facing barriers to engage in activities that foster positive youth development, which may improve socioeconomic outcomes, quality of life and long-term health status in this population. This paper outlines the study protocol measuring impact of an urban Sport for Development facility on positive youth development in youth facing barriers.

**Methods/design:**

Participants aged 6–29 will be recruited from programs at an urban Sport for Development facility to a 2-year prospective longitudinal mixed-methods study. Participants will be stratified by age into three cohorts with age-specific outcomes. Age-specific positive youth development outcomes will be assessed quantitatively by self-report and pedometer at baseline and after 6 months, 1-year, and 2-years of program participation. Focus groups will provide data regarding youth experience and the impact of facility and program components on youth outcomes.

**Discussion:**

Our findings will inform best-practice and feasibility of a Sport for Development facility delivering programs in a socially and economically challenged urban setting in a high-income country.

**Trial registration:**

ISRCTN67016999. Date of registration: October 22, 2019.

## Background

Significant barriers to positive development during youth including low socioeconomic status and other social marginalization can have negative consequences persisting into adulthood [1–4]. Physiological consequences of a stressful environment during youth confer increased risk of chronic stress related illnesses such as obesity, diabetes, cancer, and heart disease [5]. Similarly, adversity during childhood can impact the development of psychological and social functioning, further contributing to negative consequences during adulthood [6]. Persisting negative health and functional outcomes may impair attainment in other life domains such as academic, occupational, and civic engagement, limiting socioeconomic and quality of life potential and perpetuating a cycle of marginalization [7, 8]. The increased risk of poor health outcomes conferred by barriers faced during youth may be further compounded by self-regulatory problems which reduce the likelihood of engaging in healthy risk-mitigating behaviours and/or accessing appropriate care [9], while increasing the likelihood of engaging in risky behaviours [10–12].

A subpopulation of adults who faced significant barriers to positive development during youth develop no measurable negative consequences despite the adversity they have faced during childhood and are said to be “resilient” [5]. The quality of resilience is in part predetermined by genetics and psychobiological traits but may also be fostered through physical and psychosocial intervention [5]. Specifically, programs including physical activity, development of meaningful social connections, and deliberate cultivation of life skills are thought to be important factors for cultivating resilience in youth facing barriers [12–14], and may be protective against increased health and functional risk in this population.

Positive Youth Development (PYD) includes the promotion of personal characteristics described as life skills that enable individuals ‘to lead a healthy, satisfying and productive life’ and can be intentionally incorporated into sport programming [15, 16]. The development of personal assets through PYD sport programming supports an individual’s ability to engage in resilience-promoting behaviour. These positive skills and traits are transferable into adulthood and facilitate a person’s ability to engage in school and employment and participate meaningfully in social and cultural activities [8, 17], producing change that can be expected to contribute to health, social, and economic benefits on a broader social scale. In this way, PYD sport programming aimed at affecting health and function on an individual level also has implications for positive social change on a group or community level, which is more traditionally considered an objective of Sport for Development (SFD) programs. Typically, SFD and PYD remain distinct as fields of study, and the relationships between SFD and PYD through sport are not well defined, possibly due to general differences in populations and contexts of study (developed countries vs. underdeveloped countries; recreational activity vs. competitive sport setting). For an urban youth population at risk of adverse outcomes based on their developmental environment and experiences, programming designed to enhance personal assets and create social impact through intentional sport programming can achieve both PYD and SFD outcomes. This type of programming can be considered a public health intervention, as both PYD and SFD are expected to mitigate risk of adverse health and functional outcomes in youth facing barriers [9, 18, 19].

Meaningful outcomes achieved through intentional sport programming may be mediated by the PYD context, positive relationships, and explicit inclusion of life skills programming within physical activity-centred programming [18, 20–23]. Foundational to positive outcomes of sport programming is the physical and psychological safety of the PYD context [21, 24, 25], necessary for youth to engage fully. Further, relationships between youth and leaders in which leaders are relatable and act as role models, engaging youth to understand and address their individual needs to reach goals with a focus on the future is critical for uptake of programmatic intentions [20, 24, 26]. Leaders can also support development of peer-peer connections to foster a sense of belonging that contributes to both a sense of psychological safety and engagement and retention of youth participants [21]. Programs must have an explicit program theory with an intentional design that is appropriately suited to achieve the desired PYD or SFD outcomes, such as increased life skills and good health behaviours to support academic or occupational achievement [16, 24, 26–28]. Lastly, in sport-based programs, physical activity aimed at promoting the competence, confidence, knowledge, and motivation needed to engage in physical activity for life (termed ‘physical literacy’) [29] and explicit teaching of life skills using sport or physical activity participation as a learning context [20, 26, 27] are most suited for developing PYD assets that may be expected to lead to SFD outcomes.

SFD programs have demonstrated a range of benefits to youth facing barriers in a variety of studies conducted internationally, often assessing participation in a single short-term program; long-term longitudinal research is lacking. The programs studied also tend to lack use of a logic model or theory of change to explain the factors expected to lead to the attainment of the program’s objectives, whether PYD, SFD, or otherwise. Similarly, it may be expected that research on PYD through sport as evaluated by the transference of life skills through team or competitive sport participation may promote SFD and PYD outcomes in a different manner or to a different degree than community-based participatory programs. As such, the impact of a sustainable urban SFD facility promoting PYD through recreational sport plus (i.e. programs whose main objective is increased participation in sport and reducing barriers to entry to sport, but that also use sport to address broader social issues; secondary benefits can include development of life skills, education, and increased health) and plus sport (i.e. programs primarily designed to address social issues including health, education and employment that use sport as a tool to achieve some of their objectives) programming where youth may engage in several ongoing programs in addition to wrap-around services such as counselling, nutrition, and homework programs, is unknown. Further, a thorough understanding of the contextual factors of such a program and its participants is lacking, resulting in a paucity of evidence to inform best-practice for SFD and PYD through sport programs to optimize individual and community impact.

**Fig. 1:**
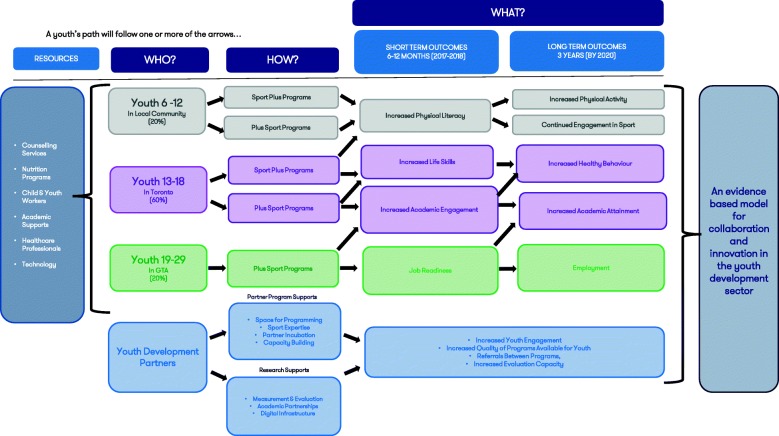
The MLSE LaunchPad Theory of Change illustrates the expected outcomes of programs for the different age groups of participants in the short- and long-term, and potential pathways for participants to follow. Supportive resources available to participants at the facility in addition to programs are indicated; additional resources may be added throughout the study duration and will be described in future publications as required. The respective cohorts are represented by age group with expected geographical catchment area and resource allocation indicated. The type of programming expected to produce the intended outcomes for each cohort are listed. This Theory of Change is expected to present a possible evidence-based model for collaboration and innovation in the youth development sector

A large, long-term longitudinal study investigating PYD outcomes in a SFD facility for urban youth facing barriers that is based on a clear theory of change illustrating program components is therefore needed. This research is important for both theoretical and practical reasons, and addresses several future research directives published in the field including calls for mixed-methods, longitudinal, and evaluative studies that validate the development of life skills through sport and evaluate the impact of program type of PYD outcomes [26, 30–32]. The current study proposes that participation in theory-based SFD programming at a dedicated facility will lead to increased PYD outcomes in urban youth facing barriers to positive development with age-specific objectives outlined in Table 1 and further articulated in Fig. 1, the Maple Leaf Sports and Entertainment (MLSE) LaunchPad Theory of Change. Repeated measures ANOVAs will be used to explore the effect of long-term participation in MLSE LaunchPad programming on age-specific outcomes. Moderation analyses will be used to identify the impact of program dosage on the relationship between time and primary outcomes. Finally, regression analyses will be used to identify the predictors of primary outcomes to better inform programming.

**Table 1:** Study Hypotheses

Primary Hypotheses	
We hypothesize that participation in MLSE LaunchPad programming and wrap around services will result in:	
a) For youth aged 6–12:	• Increased physical literacy • Increased physical activity [28] • Increased rates of continued participation in sport or physical activity [33]
b) For youth aged 12–18:	• Increased positive health behaviors relating to physical activity, sleep, nutrition, smoking, substance use and sexual activity [29, 34] • Increased life skills including critical thinking, resilience, self-esteem, self-regulation, social competence and grit [26] • Increase academic outcomes including school attendance, academic performance and high school graduation rates [26, 34] • Increased rates of continued participation in sport or physical activity [34]
c) For youth aged 19–29:	• Increased rates of placement in employment, apprenticeship, or continued training [26, 29] • Increased personal income • Increased rates of continued participation in sport or physical activity [34, 35]
Secondary Hypotheses	
We hypothesize that a) volume of participation and b) duration of participation will correlate positively with the primary outcomes listed above.

## Methods/design

### Study design

The MLSE LaunchPad trial employs a single-arm, non-randomized within subject quasi-experimental design following a cohort of 350 participants aged 6–29 years old over up to 2-years of self-selected participation in programming at MLSE LaunchPad, a Sport for Development facility in downtown Toronto, ON, Canada. Data collection methods utilize repeated measures of age-specific outcomes according to hypotheses outlined in Table 1 and further described in Table 3 using self-reports and pedometer data at baseline, 6 months, 12 months, and 24 months from baseline. Qualitative assessments will take place annually throughout the data collection period using focus groups with a subset of participants. This protocol was developed in the fall of 2016 and initiated in February 2017. These data will collectively be used to evaluate the effects of intentional sport programming in a community hub on PYD and SFD outcomes in urban youth facing barriers. Moderating variables including age, gender, race, household income, postal code, program type, volume of participation and duration of participation will be explored to understand possible explanatory individual and programmatic elements of significance.

### Ethics

All procedures have been approved by the Community Research Ethics Office and amendments will be reviewed and approved by this same ethics review body for scientific content and compliance with applicable research and human subject regulations. Informed consent will be obtained from participants aged 13 years and older and participant assent and parental consent will be obtained from participants under the age of 13 years prior to data collection. Consent/assent procedures will be conducted by a trained staff member conveying full assurance to prospective participants that their participation will not impact programming availability or access and is entirely voluntary. Additional consent will be obtained for all qualitative data collection processes. Informed consent processes will follow the Community Research Ethics Office guidelines, which adhere to the 2014 Tri-council Policy Statement on Ethical Conduct for Research Involving Humans and will be completed in accordance with the MLSE LaunchPad Privacy and Data Collection Policies [25].

### Study population

#### Inclusion criteria

Participants will be eligible for the study given the following criteria upon enrolment for the study:

- Age 6–29 years
- Registered member of MLSE LaunchPad
- Attending programming at MLSE LaunchPad
- Ability to speak, understand and read English
- Physically able to participate in programming with or without the use of assistive devices-Recruited within 8 weeks of commencing program participation.

MLSE LaunchPad defines ‘youth facing barriers’ as ‘youth who may need greater supports and services to reach their full potential’, including low income, racialized, Indigenous, new to Canada, LGBTQ2S, and homeless or underhoused youth; youth in care or leaving care; and youth in conflict with the law. Demographic data will be collected in self-report surveys and through member registration and used to define characteristics of the population. No formal process is used to ensure youth accessing MLSE LaunchPad’s programs meet the stated criteria for ‘youth facing barriers’, and no youth are excluded from programming based on these criteria; however, the neighborhood demographics and outreach strategy have to date resulted in program participation by youth who meet the criteria outlined.

#### Sample size

Based on the number of variables planned for analyses in each cohort (see Table 1) [43], we will recruit *n* = 150 youth ages 6–12, *n* = 100 youth ages 13–18 years of age, and n = 150 youth ages 18–29. Statistical power calculations were conducted to justify the appropriateness of the proposed study sample sizes for each age group. For youth aged 6 to 12, statistical power for the primary outcome variable, physical literacy, was analyzed using pre-post MLSE LaunchPad member data on the PLAYself Physical Literacy Assessment tool. To achieve a large effect size (Cohen’s d > = .8; MLSE LaunchPad participant mean = 1201, SD = 258.8; study group mean = 1277), the power calculation performed suggests that the proposed sample size of 150 will yield 94.9% post-hoc power. Power calculations were conducted using data previously collected at MLSE LaunchPad for primary outcome variables of interest in the 13 to 18-year-old cohort (i.e., self-esteem, grit, resilience, physical activity status, and physical activity levels). To achieve large effect sizes for these variables, the proposed sample size of 100 will yield 99.5–100% post-hoc power. Lastly, for youth aged 19 to 29, to achieve a large effect size in the primary outcome variable, employment status, the proposed sample size of 150 will yield 100% post-hoc power (power calculated using data collected at MLSE LaunchPad).

#### Recruitment

Recruitment of participants began in February 2017 and will continue through September 2019. Participants will be followed for up to two years with data collection to conclude by March 31, 2020. During program registration and within the first week of each program cycle, new participants (and parents/guardians if applicable) will be given information about the research study and the requirements of research participation. If the participant indicates interest, they (and the parent/guardian where applicable) will be taken through the consent process.

#### Study setting

All programming and data collection will occur at MLSE LaunchPad, a SFD facility for youth facing barriers in downtown Toronto, Ontario, Canada. The facility includes 3 sport courts, a rock-climbing wall, 3 classrooms, a commercial kitchen, wellness rooms for confidential services, a large atrium, a staff room, and multiple offices and storage spaces. MLSE LaunchPad’s stated focus is on achieving sustainable, wide-ranging social outcomes for youth facing barriers through a SFD approach. All programs are free of charge and the facility offers programs for youth facing barriers aged 6–29.

The facility is located on the ground floor of a subsidized housing building. The local area has a high proportion of subsidized housing and the highest density of homeless shelters in Canada [33, 34, 44]. The area exhibits high rates of poverty and is home to a large number of low income families including over 3000 youth [35, 43, 45, 46]. Approximately 40% of the population was born outside of Canada and approximately 40% are racialized individuals, with black as the predominant racialized group [35, 43, 45]. The area is also considered to have serious safety issues, with a high rate of criminal activity [47].

#### Intervention

Programs (described in Table 2) include a mix of sport plus programs, developed by an in-house team of sport programmers, and plus sport programs, several of which have been developed collaboratively with community partner organizations with similar target demographics and intended PYD impacts. The collaborative programming model engages the expertise of the aforementioned community partner organizations; academic partners with knowledge of PYD and SFD; and partners in the broader sport and physical education sectors. Each program falls under one of the following program pillars: Healthy Mind, Healthy Body, Ready for School, or Ready for Work. All programs incorporate sport and physical activity as a part of a psychologically and physically safe learning environment. An evidence-based coaching philosophy creates consistent coaching standards across programs, ensuring that life skills teaching is explicitly incorporated into all programs and that peer-peer and peer-leader relationships are emphasized. Coaches delivering programs are trained in life skills development and transference and physical literacy and fundamental movement skills by academic partners to provide high-quality, evidence-based and intentionally designed youth SFD programming based on the program theory explicated in the MLSE LaunchPad Theory of Change (see Fig. 1). Youth mentors, who are present in the facility before, during and after program times, act as resources to participants and facilitate connections with peers, leaders, and community resources. Youth mentors play a key role in the teaching of life skills by modelling positive behaviours and using teachable moments and are considered a key element of programming for all 3 age groups.

**Table 2:** Program Descriptions

Age	Available Programming	Expected Outcomes	Evaluation Tool
6–12	Learn to Play (Sport Plus) • Registered, structured 8-week program, 1x/week for 1 h • Coach led according to programming curriculum designed to build sport specific skills, general movement skills, and sport-specific knowledge • Minimal competition • Sport options: basketball, soccer, futsal, ball hockey, football, multisport, rugby, rock climbing, golf, lacrosse, dance, tennis, run club, adapted sport Open Gym (Sport Plus) • Unregistered, semi-structured sport program offered multiple times per week in 1-h blocks • Participant-directed learning that is supported by a coach to develop intrinsic motivation and autonomy, general movement skills, and participant-selected movement and/or sport skills Sport + Nutrition (Sport Plus) • Registered, structured 9-week program, 1x/week for 2 h • On-court physical activities, sports, and games of low organization, plus kitchen activities and nutritional education • Co-led by kitchen instructor and on-court coach • Designed to develop general movement skills, general kitchen skills, and understanding of health concepts (nutrition, physical activity etc.) PA Day Programming (Sport Plus) • Registered 1-day full-day program that includes sport and physical activity programming as well as off-court activities • Program runs on school holidays throughout the school year Day Camp (Sport Plus) • Registered 2-week full-day program that includes sport and physical activity programming as well as off court activities Plus Sport Program Partners • Quantum Sports Learning Association – Registered after-school academic support and athletic coaching program, 1 day/week for 2 h/session • Square Circle – registered after-school social circus program, 2 days/week for 2 h/session • New Leaf Yoga – unregistered after-school yoga program, 1 day/week for 1 h Homework Club Homework support is available from 4 to 6 pm 3 days/week	Increased Physical Literacy Increased Physical Activity Increased rates of participation in sport and physical activity	PLAYself 1-week pedometry Self-report of regular sport/physical activity participation All outcomes are also assessed through scheduled qualitative data collection
13–18	Learn to Play (Sport Plus) • Registered, structured 8-week program, 1x/week for 1 h • Coach led according to programming curriculum designed to build sport specific skills in tandem with a relevant life-skill, general movement skills, and sport-specific knowledge • Minimal competition • Sport options: basketball, soccer, futsal, ball hockey, football, multisport, rugby, rock climbing, golf, lacrosse, dance, tennis, run club, adapted sport League Play (Sport Plus) • Registered, structured program – duration is variable (5x/week for 1 week, 2x/week for 4 weeks, or 1x/week for 9 weeks) • Coach led including practice plans & game play, designed to build sport specific skills in tandem with a relevant life-skill, knowledge of sport-specific rules, tactics, and team play • Each program day includes practice time, a game, and a team meal • Sport options: basketball, soccer, ball hockey, multisport, volleyball Multi-Day School Field Trip (Plus Sport) • Structured 8-week program in collaboration with local schools • Classes attend 4 half-day sessions 1x/2 weeks • Program content is a blend of physical activity and workshop-style learning with two options: Sport + STEM (Science, Technology, Engineering and Math) and “Fuel for Fun” (nutrition and physical activity) • Concepts taught in programs are reinforced at school by the classroom teacher Boys’, Girls’, & Co-ed Leadership Camps (Plus Sport) • Registered, structured 5 day (1 week) full-day program • Coach led blend of on-court and off-court programming including sport-specific skills, general movement skills, teambuilding & leadership activities, and life skills Leaders-In-Training (Plus Sport) • Registered, structured 8-week full-day program (5 days/week) • Participants must apply • Program includes 2 weeks of job skills training and certifications, 5 weeks of coaching day camps, and 1 week of professional development field trips Open Gym (Sport Plus) • Unregistered semi-structured sport program • Participant-directed learning that is supported by a coach to develop intrinsic motivation and autonomy, general movement skills, and participant-selected movement and/or sport skills • Drop-in game play is available Plus Sport Program Partners: • Peacebuilders International – Registered restorative justice program, 3 h 1 day/week for 6 weeks; youth diverted from the court system participate in a blended off-court and on-court program that includes talking circles and complimentary physical activities as an alternative to sentencing • Pathways to Education Group Mentoring – Registered program, 3 h 1 day/week for 24 weeks; youth participate in a blend of off-court and on-court activities relating to themes such as identity and anti-oppression, communication and teamwork, and relationships • Pathways to Education Iron Chef – Registered program, 2 h 1 day/week for 8 weeks; youth participate in a blend of activities in the kitchen and on the court relating to food budgeting, food preparation, and healthy physical activity • SoCirc – Registered social circus program with a performance element, 3 h 1 day/week for 8 weeks	Increased Life Skills Increased Academic Performance Increased Health Behaviour Increased or continued sport and physical activity participation	Life Skills • Resilience (Child & Youth Resilience Measure) • Self-Esteem (Rosenberg Self-Esteem Scale) • Self-Regulation (Motivation and Self-Regulation Subscale from the School Attitudes Assessment Survey) • Social Competence (Social Competence Teen Scale) • Grit (Grit Scale) Academic Performance • Self-Reported School Attendance • Report Card • Self-Report of highest grade/level completed Health Behaviour • Healthy Behaviour Questionnaire • 1-week pedometry Self-report of regular sport/physical activity participation All outcomes are also assessed through scheduled qualitative data collection
19–29	Plus Sport Program Partners • NPower Canada – Classroom-based technical support professional training program that runs 5 half-days/week for 10 weeks with an on-court sport and activity component for 1 h/day 2 days/week • Covenant House – Kitchen-based hospitality placement training program that runs 5 full days/week for 8 weeks with an on-court sport and activity component for 1 h/day 2 days/week Open Gym (Sport Plus) • Unregistered semi-structured sport program • Participant-directed learning that is supported by a coach to develop intrinsic motivation and autonomy, general movement skills, and participant-selected movement and/or sport skills	Increased employment, apprenticeship, or training placement Increased rate of participation in sport or physical activity	Self-report of placement in job, apprenticeship, or training full- or part-time Self-report of regular sport/physical activity participation All outcomes are also assessed through scheduled qualitative data collection
All Ages	Events • One-time events providing specific demographic groups with novel experiences with specific intended outcomes, such as: - Empowering Girls through Sport - Kyle Lowry/Adidas event - Michael B. Jordan appearance - Community Celebration days (facility launch and anniversary celebrations) - Toronto Maple Leafs Prospect Camp - Toronto Maple Leafs “Next Gen” game - Toronto Maple Leafs and Toronto Raptors viewing parties - Basketball clinics - 3 on 3 basketball tournaments - MLSE game experiences Drop-in Counselling • Drop-in counselling with Child and Family Therapists • Therapists are available several hours each week in an on-site Wellness Room Snack Program • Fresh after-school snacks are provided 3 days/week for participants and their families	Outcomes relate to age-specific outcomes identified above	n/a

#### Core programming

Participants aged 6–12 years will be offered sport plus programs emphasizing physical literacy through a range of activity options. After-school and weekend programs including ball hockey, basketball, multi-sport, soccer, dance, and rock climbing will provide a variety of sports to act as a hook and context for PYD. These programs will have a similar structure and will include dynamic fundamental movements skills, skill and game/performance content specific to the sport, and a cool down and reflection activity. Teaching of life skills will occur through role modelling, discussion, purposefully designed program activities, incentivization and recognition, and the use of teachable moments. Some programs (i.e. multi-sport, ball hockey, football, basketball) will be offered in both co-ed and girls-only formats. The priority of this age group will be the Healthy Body program pillar with the intent to develop physical literacy as PYD asset, allowing for continued engagement in physical activity and sport as youth age out of these programs and setting the stage for additional life skills development.

Participants aged 13–18 years will be offered sport plus programs emphasizing life skills development, including multi-sport, fitness, rock climbing, dance, volleyball, ball hockey, and basketball. Plus sport programs developed in collaboration with local community organizations with demonstrated histories of positive youth impact relevant to the four program pillars will also be offered, including group mentoring programs, a restorative justice program, a kitchen skills-building program, and a social circus program. School day programs for this age group will involve weekly visits by classes from local public schools, and will focus on physical literacy and nutrition education, and exposure to Science, Technology, Engineering and Math topics on-court in a sport setting. All programs will include program-specific sport and life-skill components to achieve the objectives identified for this cohort. Some programs (i.e. basketball, volleyball) will be offered in both co-ed and girls-only programming. The priority for this age group will be the Healthy Mind and Ready for School Pillars, with a secondary emphasis on Healthy Body.

Programs for the 6–12 and 13–18 year-old cohorts will be delivered in 8-week cycles with 5 cycles each year. As research participants may participate in multiple recreational programs concurrently, volume (total program hours) and duration (number of program weeks) of participation will be used as mediating variables in analyses to examine the dose-response on objectives outlined in Table 1. All programs will include life skills development using coaching strategies discussed by Camiré et al. (2011) [19], focused on 7 life skills identified in the MLSE LaunchPad Theory of Change: grit, resilience, self-esteem, self-regulation, critical thinking, social competence, and leadership.

Participants aged 19–29 years will be offered plus sport programming developed in collaboration with local community organizations offering occupational skills training and other employment services. These agencies will demonstrate strong histories of positive youth impact and will work with MLSE LaunchPad’s sport programmers to deliver integrated employment training and sport/physical activity programming. The addition of sport and physical activity is expected to offer a different context for reiteration of in-class themes (i.e. leadership and other key life skills) and to encourage increased long-term physical activity participation. The priority of this age group will be the Ready for Work pillar. Secondary emphasis will be on the Healthy Body and Healthy Mind pillars. Due to the training nature of these programs, the format will differ from the programs offered for youth aged 6–18, and programs will generally be delivered for 4 or more hours per day, 5 days per week for 8–10 weeks, with sport programming delivered in 1-h sessions 2 days/week.

#### Wrap-around services

In addition to the programs described above, participants will be offered access to mental health counselling services, nutrition programming (healthy after school snacks offered 3 days per week during program hours), and academic assistance provided through a homework club. Structured drop-in style sport plus programs incorporating life skills teaching will also be offered year-round as an additional engagement opportunity for youth who participate in registered programs, and as an alternative for youth who are unable to commit to regular registered programming.

#### Outcomes

Participants will complete a baseline assessment within 2 weeks of recruitment. Follow-up research assessments will take place at 6 months, 12 months, and 24 months from the baseline assessment with data collection concluding March 31, 2020. Outcomes have been identified based on the program theory illustrated in the MLSE LaunchPad Theory of Change (See Fig. 1). Outcomes and indicators for the primary objectives are detailed in Table 3.

**Table 3:** Outcomes for Primary Objectives

Participants aged 6–12	
a) Increased physical literacy	Indicator:
	PLAYself Physical Literacy Assessment for Youth [36] 22-item graded self-report scale used to evaluate level of physical literacy
b) Physical activity minutes/day	Indicator:
	One-week pedometry producing at least 3 days of valid data with a minimum wear time of 10 h/day
c) Continued engagement in sport	Indicator:
	Self-report of regular sport/physical activity participation
Participants aged 12–18	
a) Life skills:	Indicator:
i. Critical Thinking ii. Resilience iii. Self-esteem iv. Self-regulation v. Social Competence vi. Grit	Critical Thinking in Everyday Life Scale [37] 20-item self-report Likert scale used to measure use of critical thinking skills including reasoning, enquiry, analysis/information processing, flexibility, and evaluation Child & Youth Resilience Measure [38] 12-item self-report scale used to measure ability to sustain well-being Rosenberg Self Esteem Scale [39] 10-item self-report Likert scale used to assess global self-esteem Motivation and Self-Regulation Subscale; derived from the School Attitudes Assessment Survey [40] 4-item self-report Likert scale used to measure ability to initiate and continue behaviours required to achieve academic goals Social Competence Scale for Teenagers [41] 9-item self-report scale used to measure positive social skills necessary to get along well with others and function constructively in groups Grit Scale [42] 8-item self-report scale used to measure stamina in dimensions of effort and interest
b) Academic Performance:	Indicator:
i. School Attendance ii. Academic Performance iii Academic Attainment	Self-report of number of missed school days over past two weeks measured as a percentage GPA as per last report card; failed courses marked as “R” on TDSB report cards will be input as 45% Self-report of highest grade/level completed
c) Physical Outcomes	Indicator:
i. Physical activity minutes/day ii. Health Behaviours iii. Continued engagement in sport	One-week pedometry producing at least 3 days of valid data with a minimum wear time of 10 h/day Healthy Behaviour Questionnaire 6-item, author-designed scale used to measure behaviours relating to sleep, nutrition, physical activity, smoking, substance use, and risky sexual behaviour Self-report of regular sport/physical activity participation
Participants aged 18–29	
a) Employment	Indicator:
i. Employment Status ii. Personal Income	Self-report of placement in job, apprenticeship or training; full- or part-time Self-report of personal income level
b) Sport Participation	Indicator:
i. Continued engagement in sport	Self-report of regular sport/physical activity participation

#### Data collection

Self-report questionnaires will be completed electronically on a personal computer, mobile phone or tablet device, or in the facility using a tablet device (Samsung Tab, iPad, or Microsoft Surface) provided by MLSE LaunchPad. Participants under the age of 8 or requiring assistance will be assisted by an adult. Participants aged 8 and over may complete questionnaires independently, but staff assistance will be offered.

Pedometer data will be collected using PiezoRx pedometers for 1 week at each research assessment time-point according to manufacturer’s instructions. Participants will be instructed on how to attach the pedometer to the waist line of clothing, to not open or tamper with the case, and to wear the device at all times unless sleeping or in water. When the pedometer is returned after 7 days, the data will be uploaded to an electronic database using minutes of activity in the Moderate to Vigorous range based on the criterion of 100 steps per minute outlined by PiezoRx.

Focus groups will be conducted annually with participants to gather personal perspectives regarding experience of programs and facility processes and impact of program involvement. Discussions will focus on: the impact of participation on health behaviours, life skills, academic engagement, job readiness and employment, and other impacts not explicitly expected or defined; the trajectory of involvement and access to programs, and wrap-around services offered in the facility.

#### Incentivization

As attendance in programs is expected to mediate anticipated outcomes of participation in programs, strategies will be used to increase youth engagement and facilitate ongoing, regular participation in registered programs. The use of a gamified digital infrastructure to incentivise target behaviours such as attendance and provide external motivation to complete research requirements will be used. A customized mobile website will centralize incentivization of program participation and research-related activities and points will be allocated for various behaviours such as attendance, early registration, completion of research questionnaires, and demonstration of positive health behaviours and key life skills. The points earned can be exchanged for prizes of the participant’s choosing from a selection of sporting goods, apparel and school supplies.

This method will allow the research team to communicate with participants remotely, collect relevant participation data centrally (i.e. program hours and attendance), and provide participants with a consistent means of accessing information and reminders for participation in both programs and research activities. We expect this strategy will engage participants through competition and goal setting and will also allow the research team to reinforce positive health behaviours and life skills included in program curricula remotely to support development and retainment of PYD assets. Further, we expect the electronic collection of data will improve protocol adherence, data accuracy, user acceptability, and adherence to timelines. Given the potential barrier regarding internet and device access, tablet kiosks will be available in the facility’s atrium to ensure all participants have access to the digital infrastructure and incentive strategy.

#### Data management

Standardized electronic capture forms will be used for all data collection to ensure clarity and ease of entry. All data will be entered electronically and exported into an SPSS (IBM Corp., New York, USA) database on a secure server at MLSE LaunchPad. Participant data will be delinked and accessed only by research staff. All data will be hosted on a secure server that is routinely serviced and backed up by MLSE IT staff. Informed consent forms will be scanned and stored on the server and hard copy documents will be destroyed upon conversion to electronic formatting. The data will be retained indefinitely. Peer audit will be conducted by research staff including research analyst, coordinator, manager, and director who will audit procedures and existing data on a cyclical basis.

#### Data analysis

Preliminary analyses will be conducted after 6, 12, and 24 months of recruitment. All statistical analyses will be completed using either SPSS versions 24/25 (IBM) or *R*. Missingness in data will be evaluated for amount of missing data and patterns in missingness. Based on the results of this evaluation, a single mean value imputation method will be applied as needed.

*Ages 6–12:* Repeated measures ANOVAs will be used to examine the main effect of time on physical literacy scores, self-reported physical activity and rates of continued participation in physical activity, controlling for demographic variables such as age and gender. Moderation analyses will be used to explore the effect of program dosage in terms of the number of programs attended over the course of participation in this study on the relationship between time and change in physical literacy. A similar moderation effect will be explored in the relationship between time and the change in self-reported physical activity levels. Pearson’s product-moment correlation coefficients will also be used to explore the relationship between physical literacy and physical activity levels. A standard multiple regression controlling for age, gender, household income and housing status will be used to identify predictive variables for each of the three primary outcomes. Potential predictors for the change in physical literacy include program dosage, type of program involvement (Sport Plus or Plus Sport), physical literacy score at baseline and physical activity level. Potential predictors for the change in physical activity level and rate of continued participation in sport or physical activity include program dosage, type of program involvement, physical activity level at baseline and physical literacy score.

*Ages 13–18:* Repeated measures ANOVAs will be used to examine the main effect of time on positive health behaviours and each of the six life skills, controlling for demographic variables such as age, gender, household income and race. Moderation analyses will be used to explore the impact of program dosage on the relationship between time and each of the six life skills. Pearson’s product-moment correlation coefficients will also be used to explore the relationship between each of the life skills, positive health behaviors and academic outcomes. Logistic regression will be used to identify predictors of high school graduation for the subset of this age group that would be expected to graduate during the study with possible predictors including scores for each life skill at 2-year follow-up, program dosage and program type, while controlling for gender, household income, housing status and baseline life skills score. The same variables will also be used in a standard multiple regression to identify predictors of continued participation in sport and physical activity in this age group.

*Ages 19–29:* Repeated measures ANOVAs will be used to examine the main effect of time on rate of employment, apprenticeship, or continued training, personal income, and rate of continued participation in physical activity, controlling for demographic variables such as age, education level, household income at baseline and housing status at baseline. Logistic regression will be used to identify predictors of employment status at 2-year follow-up, which may include program type and program dosage (number of programming hours), while controlling for similar demographic variables as identified above for the Repeated Measures ANOVA.

*Qualitative:* Focus groups will be recorded digitally for review and transcription. The transcripts will be coded manually and analyzed using a hermeneutic approach, a cyclical process in which the researcher moves between working with the entire text and smaller parts of the text. This approach educes understanding of the transcribed material by bringing forth existing suppositions and recognizes that the interpreter’s own thoughts play a role in re-awakening the texts’ meaning. Using this approach, the perspectives of MLSE LaunchPad members and staff will be understood and contextualized with reference to PYD and SFD theory through repeated reading of the text and interpretation of the phenomena described. Regular research team meetings will be held to confer on analysis and discuss emergent qualitative findings. Analyzed quantitative and qualitative data will be integrated in reporting and presentation of findings.

#### Harms

Adverse events including physical injuries will be monitored by MLSE LaunchPad’s staff team and documented through the facility’s incident reporting process.

## Discussion

The paper reviews the rationale and methodology for a 2-year longitudinal study of a community-based Sport For Development facility in an urban setting using blended PYD and SFD program theory and outcomes with a unifying Theory of Change. Mixed methods of quantitative and qualitative data collection including validated questionnaires, pedometers and focus groups will be used to measure the impact of a community-based SFD program. Age-specific outcomes related to PYD including personal, social, and physical domains [8, 23, 48] will be used to assess the impact of the facility and a variety of sport plus and plus sport programs offered to youth facing barriers to inform best practice in program model, delivery, and measurement. This facility may be uniquely positioned to develop best in class programming; it includes ongoing measurement and evaluation infrastructure and the hub model incorporates input from in-house programmers and researchers and academic, community, and sport sector partners, facilitating a bridge between research and practice in-house and within the broader SFD and PYD sectors.

Youth facing barriers to positive development have a higher risk of experiencing physical, mental, behavioural, and psychosocial challenges that may persist into adulthood. As such the adversely affected development of these critical life domains can inhibit academic, occupational, and social attainment, physical and mental health, and overall quality of life [49–53]. SFD programs including a PYD emphasis such as social, life skills, and physical mastery elements may promote resilience to broad reaching negative impacts of childhood adversity, conferring enhanced PYD to participants [5, 27]. The literature to date indicates that for SFD or PYD through sport programming to impart these lasting effects to participants, it must expand, extend, and enhance opportunities for physical activity participation [24] to explicitly teach life skills through youth sport programming [19, 25]. Further, positive and meaningful relationships with leaders/coaches and peers [19, 20, 54], personal connection to the programming [26], development of competence in activities [26], and use of a program theory [20, 23] to intentionally develop life skills using sport as a context rather than a stand-alone intervention are requisite program elements to impart PYD and SFD outcomes for youth participants.

The SFD programs described herein will incorporate these necessary elements of a successful SFD program offering intentional programming with high quality coaching, equipment, facilities and support staff that this population would otherwise not have access to. Beyond providing more numerous, varied, and quality sport opportunities for socially marginalized youth, barriers to participation are addressed through the use of youth voice and a novel digital infrastructure to incentivize continued and engaged participation that emphasizes process-oriented goal pursuit to enhance personal investment in the program and opportunities for supplementary PYD and life-skill development through mobile access of program information and research activities. Intentional programming emphasizing age-specific life skills transference in a sport context with wrap-around support services will be used to prioritise physical literacy and activity in youth aged 6–12, academic engagement and positive health behaviours including physical activity in youth aged 13–18, and occupational readiness and physical activity habits in youth aged 19–29. The age-specific prioritization of objectives is designed to provide participants with the necessary life skills to reach their potential socially, physically, and psychologically in order to engage in academic, occupational, and civic pursuits, ultimately improving broader social and economic outcomes in this currently marginalized urban community in Toronto, Ontario, Canada.

To the authors’ knowledge, this is the first long-term longitudinal study of SFD programming in an urban setting using both quantitative and qualitative methods of data collection and analysis. We believe that this program is uniquely positioned to deliver best in class SFD and PYD through sport programming given the collaborative development of evidence-based programming and embedded measurement and evaluation bridging the gap between research and practice. Further, the inclusion of community partners, a digital gamified delivery of participation incentivization, and branding affiliation with a local professional sports organization may help overcome several existing barriers to participation and optimize the acquisition of age-specific PYD outcomes that will lead to larger scale community impacts.

## Data Availability

The datasets analysed during the current study will be available from the corresponding author on reasonable request.

## Abbreviations

PYD: Positive Youth Development
SFD: Sport for Development
